# BRAIN RESPONSE IN THE ELDERLY AFTER TAI CHI PRACTICE ASSESSED WITH NEUROIMAGING TECHNIQUES: A LITERATURE REVIEW

**DOI:** 10.1093/geroni/igac059.2595

**Published:** 2022-12-20

**Authors:** Hao (Howe) Liu, Yasser Salem, Eric Arguello

**Affiliations:** Allen College, Waterloo, Iowa, United States; Hofstra University, Hempstead, New York, United States; Allen College, Waterloo, Iowa, United States

## Abstract

Tai Chi (TC) has been often provided to older adults by rehabilitation professionals for medical dysfunction and anti-aging healthcare. In the last 10 years, there has been an increase in the number of studies examining the effects of TC on brain as assessed by neuroimaging including near infrared spectroscopy (NIRS), and structure and functional magnetic resonating imaging (sMRI & fMRI). Thus, the purpose of this literature review is to evaluate how TC practice may affect the brain in the elderly as assessed by neuroimaging techniques. A comprehensive literature search was conducted using a variety of key words with different search engines to search from the last 10 years until January 15, 2022. Studies were included if they investigated topographic brain responses after TC practice in the elderly population. A total of 12 original studies with 15 articles met the criteria and were included for the review process. The results showed increased volume of cortical grey matter, improved neural activity, and increased neural connectivity in different brain regions, including the frontal, temporal, and occipital lobes, followed by cerebellum and thalamus. Intriguingly, the longer one practices TC, the more his/her brain areas may be altered. Such neural findings after TC practice are often associated with neurobehavioral improvements in attention, cognitive execution, memory, emotion, and risk-taking behaviors. Tai Chi is a promising exercise that is able to improve morphological capability and neurofunctional activity in the brain in the elderly. These improvements seem to be associated with time-length of TC practice.

